# Semi-automatic segmentation from intrinsically-registered 18F-FDG–PET/MRI for treatment response assessment in a breast cancer cohort: comparison to manual DCE–MRI

**DOI:** 10.1007/s10334-019-00778-8

**Published:** 2019-09-27

**Authors:** Maren Marie Sjaastad Andreassen, Pål Erik Goa, Torill Eidhammer Sjøbakk, Roja Hedayati, Hans Petter Eikesdal, Callie Deng, Agnes Østlie, Steinar Lundgren, Tone Frost Bathen, Neil Peter Jerome

**Affiliations:** 1 Department of Circulation and Medical Imaging, NTNU, Norwegian University of Science and Technology, Trondheim, Norway; 2 Department of Physics, NTNU, Norwegian University of Science and Technology, Trondheim, Norway; 3 Department of Radiology and Nuclear Medicine, St. Olav’s University Hospital, Trondheim, Norway; 4 Department of Clinical and Molecular Medicine, NTNU, Norwegian University of Science and Technology, Trondheim, Norway; 5 Department of Oncology, St. Olav’s University Hospital, Trondheim, Norway; 6 Section of Oncology, Department of Clinical Science, University of Bergen, Bergen, Norway; 7 Department of Oncology, Haukeland University Hospital, Bergen, Norway

**Keywords:** Breast cancer, Diffusion imaging, Mixture modelling, PET/MRI, Segmentation

## Abstract

**Objectives:**

To investigate the reliability of simultaneous positron emission tomography and magnetic resonance imaging (PET/MRI)-derived biomarkers using semi-automated Gaussian mixture model (GMM) segmentation on PET images, against conventional manual tumor segmentation on dynamic contrast-enhanced (DCE) images.

**Materials and methods:**

Twenty-four breast cancer patients underwent PET/MRI (following 18F-fluorodeoxyglucose (18F-FDG) injection) at baseline and during neoadjuvant treatment, yielding 53 data sets (24 untreated, 29 treated). Two-dimensional tumor segmentation was performed manually on DCE–MRI images (manual DCE) and using GMM with corresponding PET images (GMM–PET). Tumor area and mean apparent diffusion coefficient (ADC) derived from both segmentation methods were compared, and spatial overlap between the segmentations was assessed with Dice similarity coefficient and center-of-gravity displacement.

**Results:**

No significant differences were observed between mean ADC and tumor area derived from manual DCE segmentation and GMM–PET. There were strong positive correlations for tumor area and ADC derived from manual DCE and GMM–PET for untreated and treated lesions. The mean Dice score for GMM–PET was 0.770 and 0.649 for untreated and treated lesions, respectively.

**Discussion:**

Using PET/MRI, tumor area and mean ADC value estimated with a GMM–PET can replicate manual DCE tumor definition from MRI for monitoring neoadjuvant treatment response in breast cancer.

## Introduction

Breast cancer is the most frequent type of cancer in women worldwide [1], with a mean 5-year survival of 90.4% in Norway [2]. Patients diagnosed with locally advanced breast cancer (LABC, stage 3), have a worse survival outcome (78.3%) [2]. They receive neoadjuvant chemotherapy treatment before surgery with the goal of complete pathological tumor regression, which correlates with improved survival and a reduced chance of breast cancer recurrence [3]. Objective response evaluation during neoadjuvant therapy is important assess treatment efficacy and to avoid unnecessary toxic side effects [4]. Radiologically, response evaluation has traditionally focused on measurements of tumor size [5], but several recent studies [6–10] have established functional imaging modalities as useful indicators of early response during neoadjuvant chemotherapy.

Diffusion-weighted magnetic resonance imaging (DWI) is a functional imaging modality with contrast arising from water molecule motion, and is, therefore, sensitized to tissue microstructure characteristics. DWI is most commonly utilized to assess tissue cellularity, where highly cellular tissues such as malignant tumors exhibit decreased diffusivity [11], quantified by calculation of an apparent diffusion coefficient (ADC). A robust empirical biomarker that is reduced in malignant tumors [12], ADC has shown higher specificity than conventional anatomical MRI for discriminating malignant and benign breast tumors [13].

Tumor ADC is commonly measured by the mean value of manually placed regions-of-interests (ROIs). There is no standard protocol for this tumor segmentation, and different approaches can significantly influence resulting ADC values [14]. Given that direct tumor segmentation of DWI may be confounded by noise and lack of conspicuity, tumor ROIs are commonly delineated on dynamic contrast-enhanced (DCE) images before being transferred to DWI. The definition of tumor on DCE images is thus governed by leakage of gadolinium contrast through pathological vessels and, therefore, linked to vascularity, whereas diffusion changes, reflecting cellularity, do not necessarily coincide [15].

Simultaneous positron emission tomography and magnetic resonance imaging (PET/MRI) is a recent technology with a significant potential in many aspects of breast cancer practice, including diagnostics, staging, and neoadjuvant response evaluation [16]. PET/MRI examinations allow simultaneous collection of structural, functional, and metabolic imaging properties in the same spatial and temporal domain. 18F-fluorodeoxyglucose (FDG)–PET visualizes upregulated glucose metabolism, while MRI reflects other hallmarks of cancer [17] including invasion and metastatic propensity (by ADC) and increased angiogenesis (DCE). Several studies report correlations between standardized uptake values (SUV) from FDG–PET and ADC in malignant tissue [18–20], indicating that intrinsically-registered 18F-FDG uptake may provide an alternative approach to manually drawn DCE–ROI delineation for use in DWI analysis [19]. FDG–PET is also known to outperform MRI tumor volume measurements in some cancers [21]. In this study, a simple, semi-automated Gaussian mixture model (GMM) segmentation algorithm was selected, to allow for heterogeneous FDG uptake across tumors and expected decline through treatment [22, 23].

The aim of the current study is to investigate the reliability of deriving lesion diffusion imaging characteristics from 18F-FDG uptake in invasive breast cancers > 4 cm or LABC (i.e., cT2-4N0-3) during neoadjuvant treatment. Specifically, we tested the reliability of deriving functional tumor area and ADC values in diffusion-weighted images from intrinsically-registered 18F-FDG–PET uptake using a semi-automated GMM segmentation algorithm in comparison with metrics derived from manually drawn DCE–ROIs.

## Materials

### Participants

This prospective study was approved by the Regional Committee for Medical and Health Research Ethics (REC) in western Norway (identifier 2015/1493). Informed consent was obtained from all individual participants included in the study. A total of 24 patients (median age 53 years, range 37-74) with biopsy-proven, invasive breast cancers > 4 cm or LABC (i.e., cT2-4N0-3). This minimum size was an inclusion criterion for recruitment to the phase II PETREMAC trial (Clinicaltrials.gov #NCT02624973), where lesions of this size are targets for neoadjuvant chemotherapy. The patients underwent individualized neoadjuvant therapy, based on tumor characteristics: estrogen (ER)/progesterone receptor (PgR), human epidermal growth factor-2 (HER2), and TP53 mutation status. The therapy used was primarily endocrine treatment (full details given in Table 1).

**Table 1:** Clinical characteristics of patient cohort

Characteristic	All patients (*n* = 24)
Age (median, range), years	53 (37–74)
Height (median, range), m	1.65 (1.54–1.79)
Weight (median, range), kg	67 (50–100)
Tumor volume (median, range), cm^3^	9.91 (2.88–60.56)
Histological type	
IDC	18
ILC	2
Other	4
Histological grade	
1	0
2	9
3	13
Unknown	2
Estrogen receptor (ER) status (%)	
Negative	8
≥ 1–10	1
≥ 10–50	0
> 50	15
Progesteron receptor (PgR) status (%)	
Negative	9
≥ 10–50	1
≥ 50	4
HER2 status	
Negative	15
Positive	7
Not applicable	1
Ki67 (%)	
< 30%	9
≥ 30%	15
Treatment	
Endocrine	11
Docetaxel and cyclophospamid	1
Pertuzumab, trastuzumab and docetaxel/cyclophosphamid	3
Pertuzumab, trastuzumab and docetaxel	5
Olaparib and carboplatin	4
Pathological response	
Complete response	8
Non-response	13
Not operated	3

Pathological characteristics are determined based on histopathologic analysis of pre-treatment core needle biopsy; for *n* = 3 patients, histological grade was determined from surgical specimen

Others (ICD and ILC (*n* = 1), poorly differentiated carcinoma (*n* = 1), carcinoma with medullary features (*n* = 2)

*IDC* invasive ductal carcinoma, *ILC* invasive lobular carcinoma

**Fig. 1:**
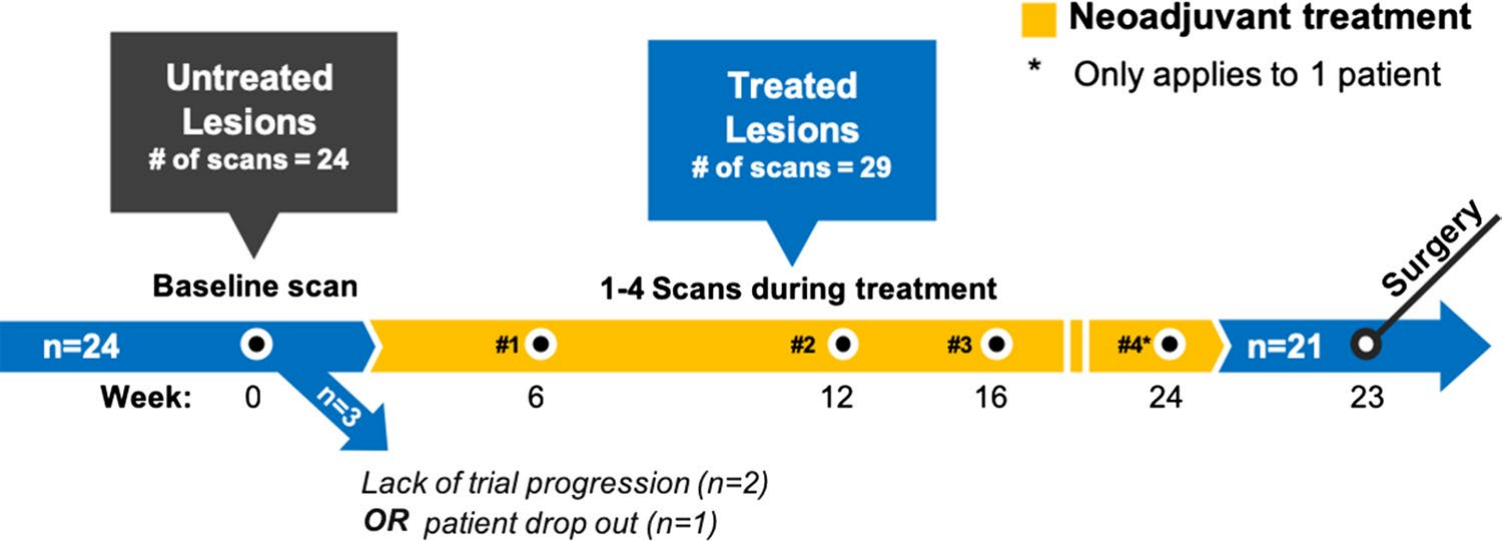
Breast cancer patients (*n* = 24) received one pretreatment baseline scan, and additional scans during neoadjuvant treatment prior to surgery. On average, scans during treatment were 6, 12, 16, and 24 weeks after baseline, and surgery was 23 weeks after baseline scan. Total data set included 53 scans: 24 from untreated, and 29 from treated lesions

Patients were examined with 18F-FDG–PET/MRI scans, at baseline and up to four scanning sessions during neoadjuvant treatment (depending on trial progression and individual response). Two patients received neoadjuvant therapy 2 days prior to the baseline scan. The cohort scans are summarized in Fig. 1. All except three patients have undergone breast surgery (one patient dropped out, two scheduled after time of analysis). Tumor categorization was done by histopathologic analysis of core needle and open incisional biopsies.

Lesions with no remaining enhancement on DCE were excluded (8 data sets), resulting in 53 data sets overall: 24 untreated lesions, and 29 of treated lesions. Median number of scans was 2 (range 1–5), with mean intervals from baseline of 6, 12, 16, and 24 weeks. The mean time from study entry to surgery was 23 weeks. The two patients receiving neoadjuvant therapy 2 days prior to the baseline scan were considered untreated, as the lesions at this timepoint had undergone minimal treatment effect. This study did not explicitly consider clinical outcome or treatment effects, and therefore, this classification is predominantly to distinguish between lesions that have had the opportunity to undergo significant response.

## Methods

### PET/MRI acquisition

All patients underwent simultaneous PET/MRI on a 3 T Biograph mMR scanner (Siemens Healthcare, Erlangen, Germany), 75 min after 18F-FDG injection (4 MBq/kg dose following 6 h fasting). The 18F-FDG was produced by the Norwegian Medical Cyclotron Center (Oslo, Norway). MRI acquisition utilized a designated 4-channel breast coil and included Dixon, T2-weighted, DWI, and DCE. DCE parameters included: 3D FLASH sequence, transverse orientation, TR/TE 5.88/2.21 ms, resolution 0.7 × 0.7 × 2.5 mm, 72 slices, flip angle 15°, 1 baseline, and 7 contrast sequences, time resolution 1 min. Multiple *b* value DWI parameters were: axial bilateral single-shot echo planar imaging, TR/TE 9000/77 ms, fat suppressed, *b* values = 0, 50, 120, 200, 400, 700 mm^2^ s^−1^, resolution 2 × 2 × 2.5 mm, 60 slices, and FoV 380 × 190 mm, with additional phase-reversed *b* = 0 mm^2^ s^−1^ (hereafter ‘b0’) image. Concurrent PET data were acquired at a bed position giving full breast region coverage, and reconstructed using a manufacturer-supplied algorithm (OSEM-PSF, 21 subsets, 3 iterations, and a 4 mm full-width-half-maximum Gaussian filter; Siemens, Erlangen). PET data concurrent with DCE acquisition were available for *n* = 31 data sets (16 untreated and 15 treated lesions).

### Image preparation and manual segmentation (Manual DCE)

**Fig. 2:**
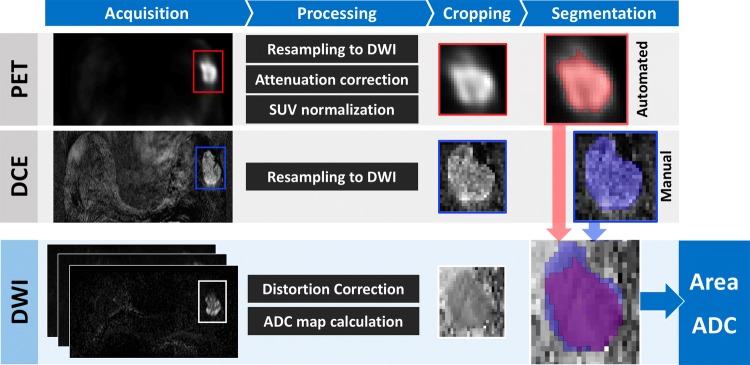
All images were resampled to diffusion-weighted imaging (DWI) resolution. PET images were attenuation-corrected and SUV normalized. Apparent diffusion coefficient (ADC) maps were calculated from distortion-corrected diffusion images. Tumor segmentation was performed by semi-automated Gaussian mixture modelling (GMM) segmentation on cropped PET images, and manually on DCE images. Resulting regions-of-interest (ROIs) were transferred to the ADC maps for derivation of tumor area and mean ADC

The lesion segmentation process is summarized in Fig. 2. DW images were distortion-corrected using phase-reversed b0 images [24]; dixon-based PET attenuation correction was performed on the scanner [25], and standardized uptake values (SUV) were normalized against body weight and dose. DCE and PET images were resampled using Elastix [26] to exactly match the resolution of the DWI images, and thus give direct voxel-to-voxel correspondence. Perfusion-insensitive ADC maps were calculated from DWI data (*b* ≥ 200 mm^2^ s^−1^) using a mono-exponential model:

$$S_{b} = S_{b = 0} \cdot {\text{e}}^{ - b \cdot ADC}$$

Manual DCE: manual segmentation of a single tumor region was performed by researcher (M.M.S.A.) on a single central slice of the enhancing solid tumor on DCE, ignoring satellite regions, with resulting ROIs supervised and approved by an expert radiologist (A.Ø.).

### Lesion cropping and Gaussian mixture modelling (GMM–PET)

A rectangular region containing the visible lesion was manually cropped from the SUV map (corresponding to the single central slice of enhancing tumor on DCE) for Gaussian mixture modelling (GMM–PET). An algorithm using default k-means++ [27] initialization (MATLAB; Mathworks, Natick, MA, USA) and an assumption of three Gaussian distribution classes were used, returning an assignment for each voxel based on highest probability [28] of belonging to each class: tumor (highest intensity), ‘non-tumor’ background (lowest intensity) and unknown (intermediate intensity). To compromise between accuracy and avoiding overestimation from partial volume effects, voxels classed as ‘unknown’ were considered non-tumor, defining the tumor class threshold as the intersection of tumor and unknown class distributions (Fig. 7 in Appendix). User input is thus limited to initial region cropping.

Two common simple thresholding-based PET segmentation methods, a fixed threshold of 2.5 (SUV_2.5_) and 42% of the maximum SUV (SUV_42%_) [22], were also performed to provide comparison with GMM–PET (Fig. 7 in Appendix).

### Derivation of DWI metrics from manual DCE and GMM–PET

ROIs, for the whole lesion within the chosen slice, derived from both manual DCE and from GMM–PET were transferred to ADC maps and used to calculate the tumor ROI area and the mean ADC value for the whole ROI.

### Statistical analysis

Performance of the three PET segmentation techniques in reference to manual DCE–ROIs was measured using the Dice similarity coefficient, varying between 0 and 1 indicating degree of spatial overlap [29], and center-of-gravity displacement (CoG). CoG was normalized based on corresponding area DCE. Tumor area and ADC values from the different segmentation methods were compared using a paired *t* test, and Pearson’s test for correlation; relationship of these segmentation metrics with SUV_2.5_ and SUV_42%_ was also assessed. A two-sample *t* test was used to assess the difference between the untreated (*n* = 24) and treated cohort (*n* = 29) for all metrics. All *p* values were corrected for multiple testing with the Benjamini and Hochberg [30] approach, with values < 0.05 considered statistically significant.

## Results

### Derived diffusion parameters (area, ADC) in GMM–PET versus manual DCE

**Fig. 3:**
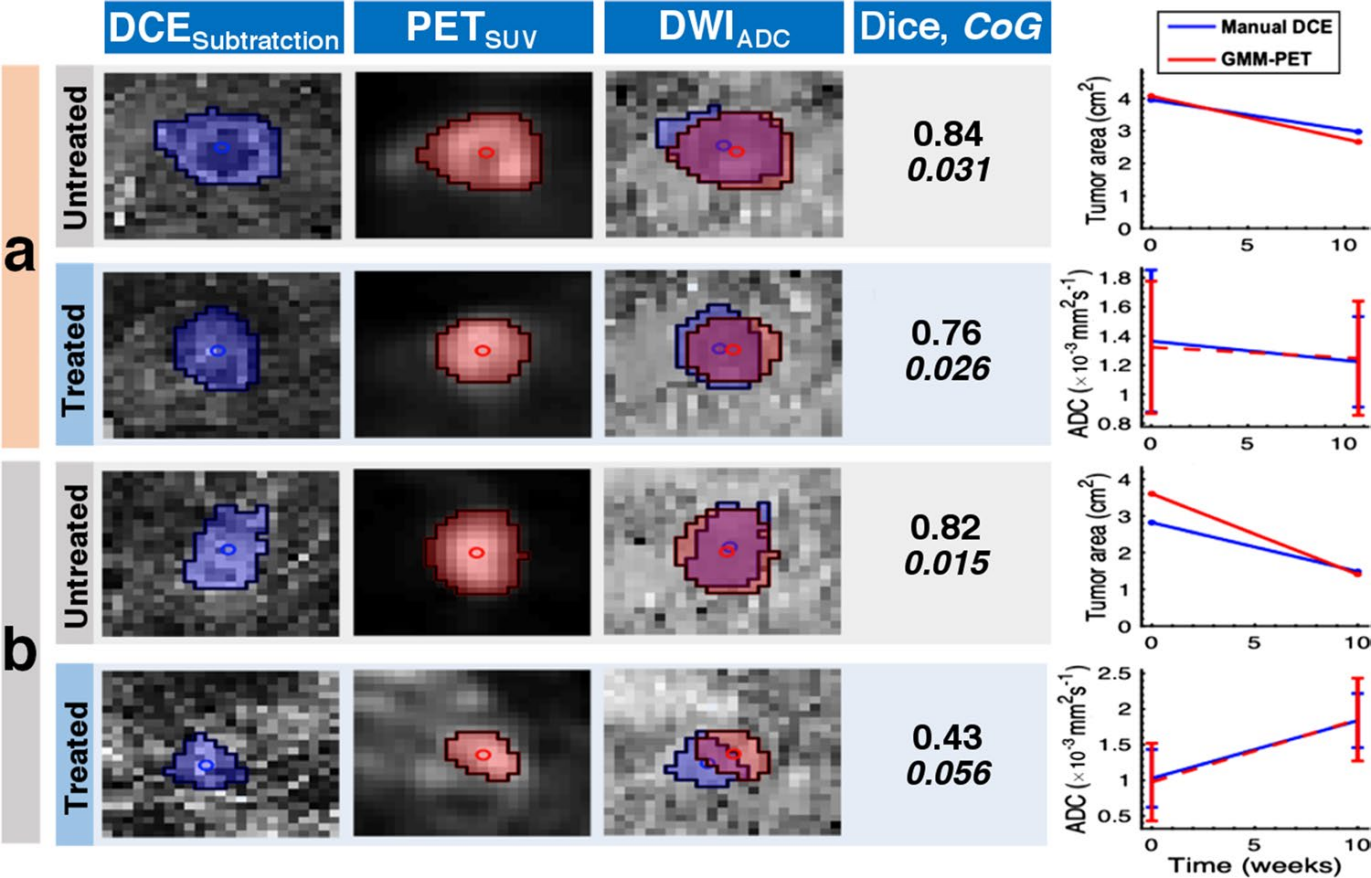
Patient in **a** demonstrated good spatial overlay and excellent agreement of response parameters over time. The patient in **b** shows a patient with excellent spatial agreement for the untreated lesion, but for the treated lesion, the segmentation is offset, with corresponding poor performance parameters (low Dice score and high CoG). However, GMM–PET was still able to accurately assess parameter changes over time

DWI metrics for untreated, treated, and all lesions are given in Table 2, and show no significant differences. There were strong positive correlations between GMM–PET and manual DCE for area and ADC for untreated and treated lesions, as shown for longitudinal scans of two patients receiving neoadjuvant therapy in Fig. 3. GMM–PET successfully tracks the same changes in ADC and tumor area observed using the manual DCE, even when performance parameters to manual DCE are poor.

**Table 2:** Values given as mean (range) and *p* values

	ADC mean (× 10^−3^ mm^2^ s^−1^)			Tumor area (cm^2^)		
	Manual DCE	GMM–PET	*p* value	Manual DCE	GMM–PET	*p* value
Untreated (*n* = 24)	0.957 (0.3796)	0.964 (0411)	0.930	6.189 (4.277)	5.923 (3.944)	0.899
Treated (*n* = 29)	1.167 (0.392)	1.1701 (0.445)	0.930	4.076 (2.857)	4.147 (2.745)	0.930
All (*n* = 53)	1.073 (0.397)	1.079 (0.439)	0.930	5.015 (3.677)	4.936 (3.416)	0.930

No significant difference between resulting parameters from manual DCE and GMM–PET

### Spatial agreement of GMM–PET with manual DCE

**Fig. 4:**
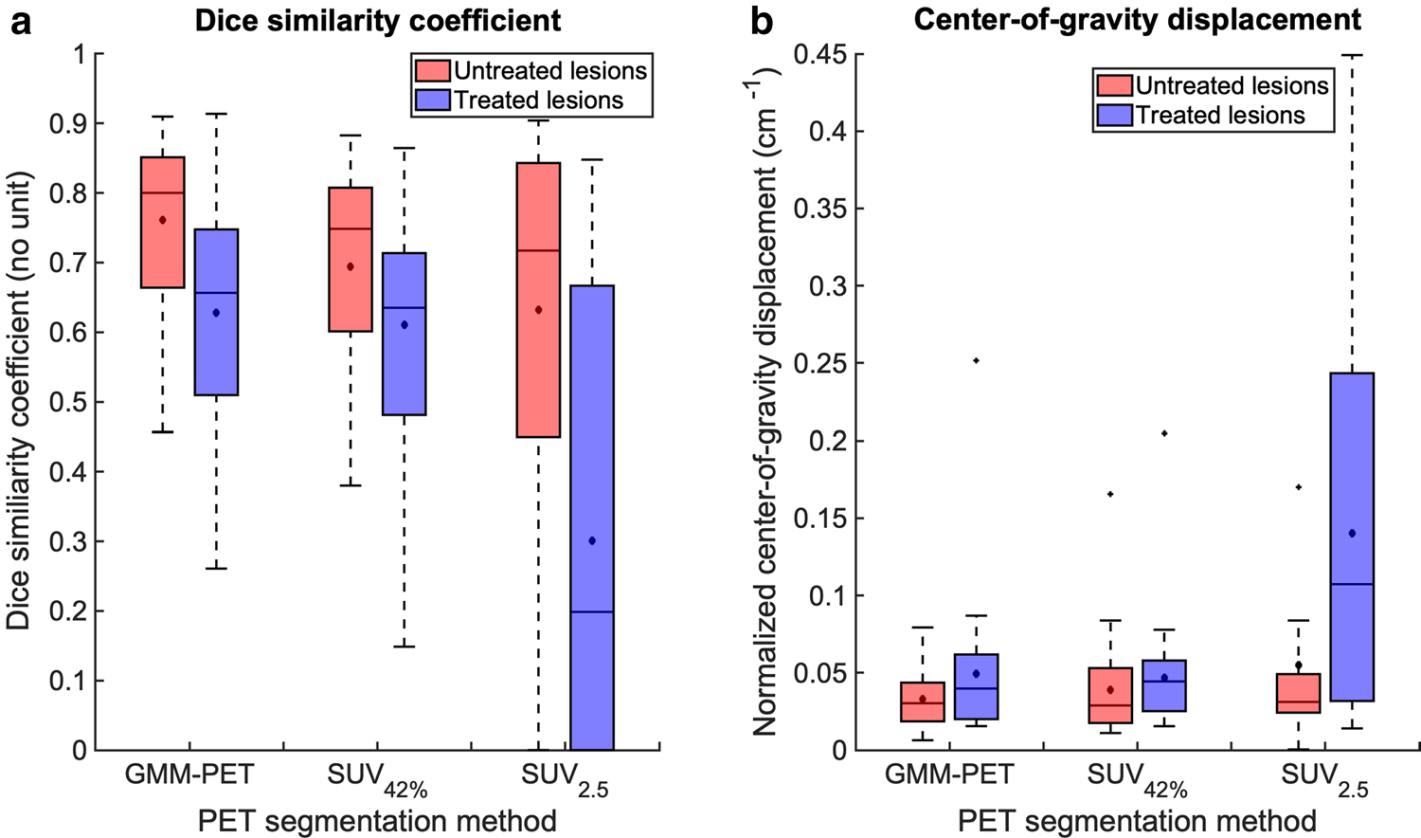
**a** Dice similarity coefficient and **b** center-of-gravity displacement, normalized to manual DCE tumor area, from GMM, SUV_42%_, and SUV_2.5_. Median and mean values indicated by lines and asterisks; boxes show interquartile range, and whiskers show data range

Dice score [29] for GMM–PET was significantly higher, indicating better performance, than SUV_42%_ for untreated lesions (*p *= 0.012) and higher than SUV_2.5_ for both untreated (*p *= 0.024) and treated lesions (*p *< 0.001) (Fig. 4a). CoG measurements were significantly lower for GMM–PET compared to SUV_2.5_ for treated lesions (*p *= 0.002) (Fig. 4b). GMM–PET is able to successfully identify tumor tissue in untreated lesions where uptake is heterogeneous across the cohort, where SUV_42%_ and SUV_2.5_ over- and underestimate tumor areas, respectively, compared to the DCE definition. In 16 cases (3 untreated, 13 treated lesions), SUV_2.5_ could not define any tumor area, meaning that CoG measurements were not applicable for these cases. GMM–PET and SUV_2.5_ performed significantly better in the treated lesions group compared to untreated lesions for both Dice score (*p *= 0.005 and *p *= 0.002) and CoG (*p *= 0.025 and *p *= 0.005), while SUV_42%_ only had significantly higher CoG (*p *= 0.002).

## Discussion

The main finding of the current breast cancer study is that functional tumor area and corresponding mean ADC values from GMM–PET ROIs matched those derived from manual DCE. As a superficial interpretation, these findings indicate that GMM–PET is a reliable technique to efficiently derive functional diffusion parameters for monitoring neoadjuvant treatment response in breast cancer. The segmentation is data driven, requiring minimal user input, and obviates the requirement for gadolinium contrast administration and, therefore, could have significant potential as an alternative objective evaluation method among the increasing number of breast cancer patients undergoing neoadjuvant treatment. At a deeper level, the results illuminate the concordance—and discordance—between ROIs derived from different imaging modalities, and as such allow interrogation of the spatial relationship existing between functional information arising from PET, diffusion, and DCE imaging, and ultimately the tissue characteristics these modalities are sensitized to.

Our study demonstrates a strong correlation between tumor ADC values derived from GMM–PET and manual DCE segmentation, in line with a previous study by Byun et al. [19] using a similar approach in breast carcinomas; our study utilizes the intrinsic voxel correspondence of simultaneous PET/MRI, thus avoiding the additional registration required by sequential FDG–PET/CT and DWI and conferring greater confidence in the results. Notably, the calculated mean ADC from GMM–PET was not significantly different from mean ADC from manual DCE, despite ADC metrics having been shown to be significantly influenced by segmentation method [14, 31]. This suggests that GMM–PET may have value even while accurate assessment of ADC metrics is considered increasingly important in a neoadjuvant treatment response setting [6–8].

Conventional manual DCE segmentation means that diffusion measurements, reflecting cellularity [13, 32, 33], are drawn from areas defined by gadolinium contrast enhancement, which is not necessarily optimal and may introduce bias to functional biomarker measurements [15]. It can thus be argued that tumor definition for diffusion studies is better performed on another MR modality more closely related to cellularity. Several studies have described an underlying link between metabolism and cellularity, such as correlation between FDG uptake to cellularity [34, 35]. Consequently, a negative correlation should be expected between SUV and ADC, and it could be argued that GMM–PET would coincide better with changes in cellularity. However, the previous reports are contradictory with either negative [18–20] or no [36, 37] correlations between SUV and ADC, indicating that imaging metrics from DCE, DWI, and PET do not capture all relevant physiological properties, even when GMM–PET is able to localize tumors equivalent to DCE.

GMM–PET segmentation gives good spatial concordance with manual DCE for untreated breast cancer lesions, while the segmentation performance was significantly poorer for treated lesions, with lower Dice score and higher CoG. It is well known that therapy affects tumor vascularity [38], which may have influenced both manual DCE and GMM–PET segmentation, as both modalities are dependent on sufficient blood flow for contrast and tracer uptake. However, DCE and PET reflect different physiological properties, and therefore, it would be of interest to observe if these have been altered differently during treatment. In addition, it should be noted that several treated lesions with poor overlap are cases where manual DCE segmentation was difficult due to low enhancement on DCE.

**Fig. 5:**
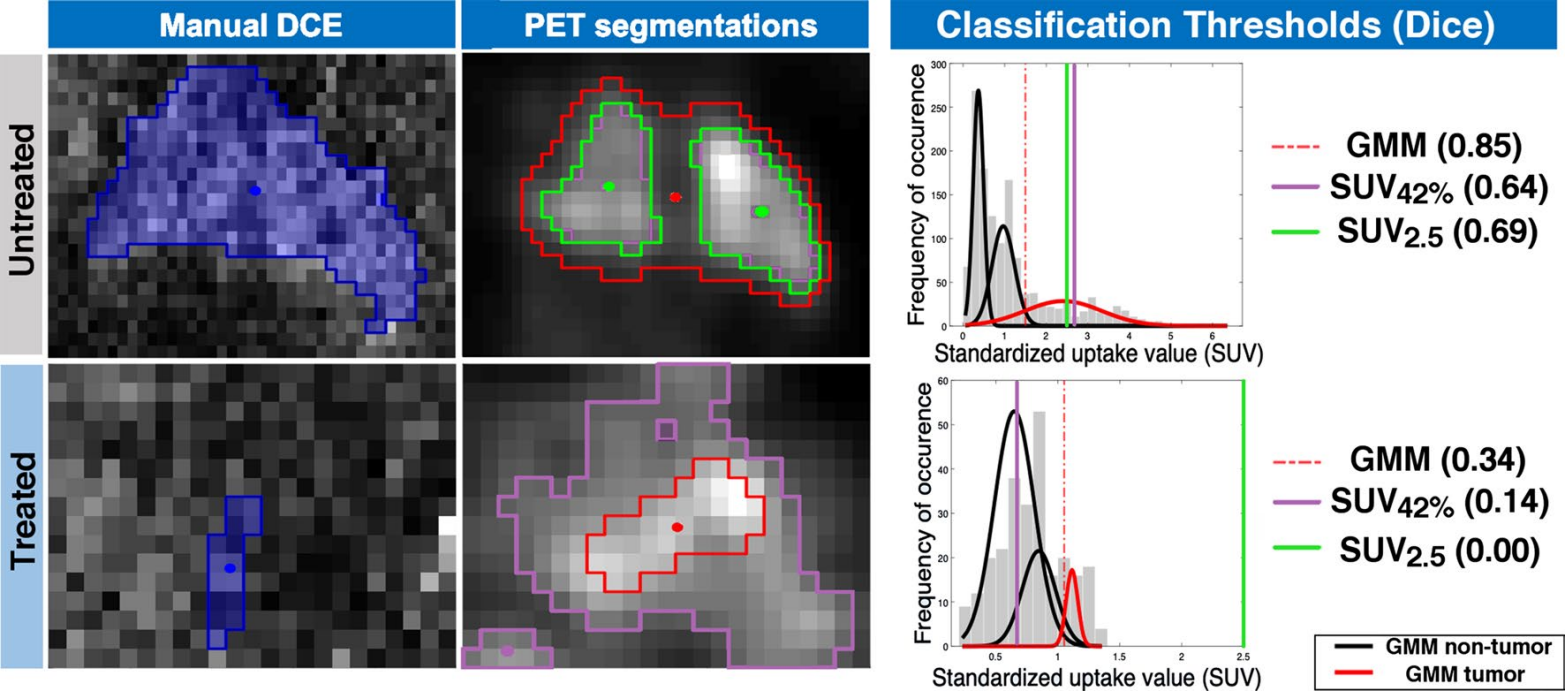
Change in GMM–PET, SUV_42%_, and SUV_2.5_ for an untreated (top row) and treated (bottom row) lesion from an illustrative patient, with corresponding histogram displays of SUV signal intensity and Dice similarity coefficient. GMM–PET is able to properly identify the whole tumor tissue of the heterogeneous untreated lesion, while SUV_2.5_ and SUV_42%_ give lower estimates. As SUV is reduced through treatment, SUV_2.5_ cannot classify any tumor tissue, SUV_42%_ overestimates tumor area relative to DCE, while GMM–PET remains stable. Using DCE as a tumor definition standard becomes problematic when contrast leakage is reduced through treatment

**Fig. 6:**
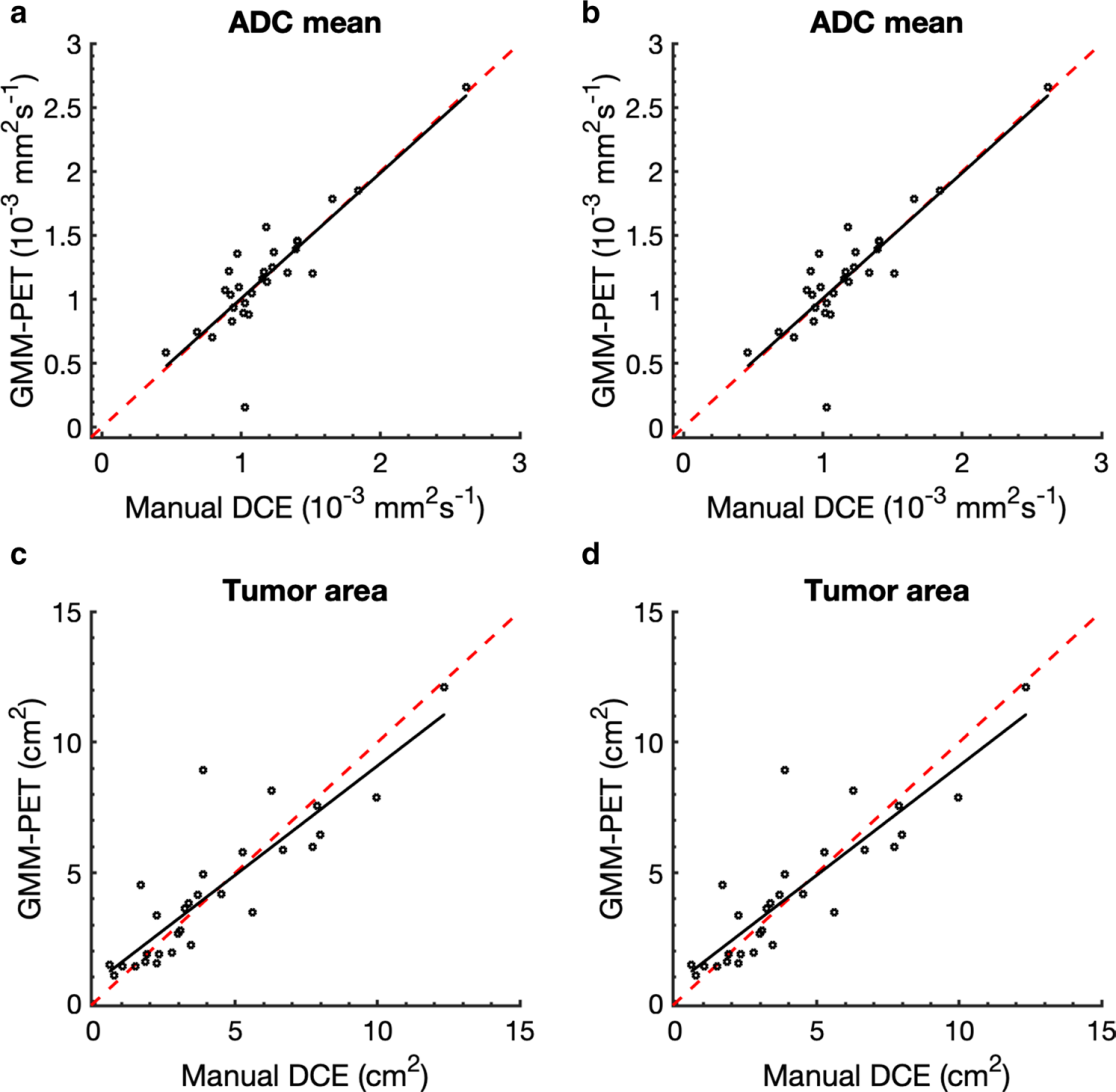
Relationship between the resulting metrics from manual DCE and GMM–PET for **a** ADC mean for untreated lesions (*r* = 0.866, *p *< 0.001) and **b** treated lesions (*r* = 0.895, *p *< 0.001) and m tumor area from **c** untreated (*r* = 0.870, *p *< 0.0001) and **d** treated (*r* = 0.928, *p *< 0.001) lesions. Red identity lines included show that area from GMM–PET is slightly smaller than from manual DCE

**Fig. 7:**
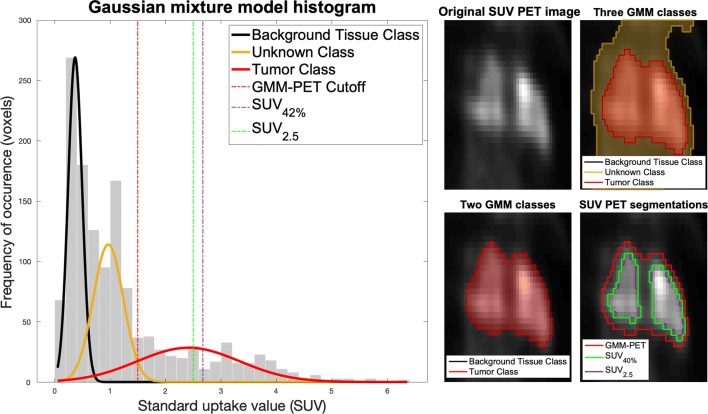
Schematic illustration of the semi-automated Gaussian mixture model (GMM) segmentation performance, and the thresholding segmentation methods SUV_42%_ and SUV_2.5_. **a** Histogram display of the three Gaussian distributions, together with SUV_42%_ and SUV_2.5_ thresholds. **b** Cropped original PET image (upper), and resulting areas from GMM segmentation algorithm (lower) with three classes: tumor (red), unknown (yellow), and background tissue (black) class. **c** GMM segmentation (red), SUV_42%_ (blue), and SUV_2.5_ (green) ROIs overlaid on original PET image. For this example, SUV_42%_ is similar to GMM, but SUV_2.5_ provides a much lower estimate of tumor volume

In this study, GMM–PET segmentation performed significantly better than the commonly used SUV_42%_ threshold in untreated lesions, and SUV_2.5_ for both treated and untreated lesions in recapitulating manual DCE. Using GMM–PET, the tumor area is not underestimated in the cases of heterogenous uptake (Fig. 5) or for tumors with high-intensity relative to their immediate surroundings, which is a well-known issue [39, 40]. However, as FDG uptake in the tumor decreases during treatment [23], GMM–PET did not perform better than SUV_42%_, which is known to give larger estimates of tumor size as SUV_max_ approaches background levels (Fig. 5), which is a limit of adaptive and data-driven algorithms that are sensitive to the FDG-uptake range [41, 42]. Other algorithm-based approaches such as gradient methods [40, 43] have also been recommended [22], although these require increased user input and were not investigated in this study (Fig. 6).

While the current findings suggest that GMM–PET segmentation can work as a proxy for manual DCE, we do not suggest that PET might replace manual DCE in today’s clinical context. Manual DCE is currently the most sensitive test for both breast cancer detection [44] and monitoring treatment response in a neoadjuvant setting [45], although concerns regarding contrast allergy and potential brain deposition [46] of gadolinium create a setting for exploration of complementary techniques. The use of PET tracer comes with its own challenges, in handling and cost, and is neither available nor suitable in all contexts. Our study indicates that the use of PET data for tumor segmentation is more reliable in pre-treatment lesions; in cases where FDG uptake is substantially reduced by treatment the GMM–PET method becomes less effective, where the tumor may become more diffuse. In these cases, automated segmentation procedures will be more prone to variation. It is worth noting that this is not unique to the technique in this study; the reduction of DCE contrast in successfully treated tumors also makes tumor definition more challenging for the conventional approach. Thus, in a simple sense, PET data are able to act as a proxy for tumor definition using DCE, but are also more likely to become useful as an adjunct to DCE for deeper investigations of tumor characteristics in multimodality examinations. It would also be possible to examine other modality combinations, such as taking a PET ROI definition to derive quantitative DCE markers from a suitable protocol.

**Fig. 8:**
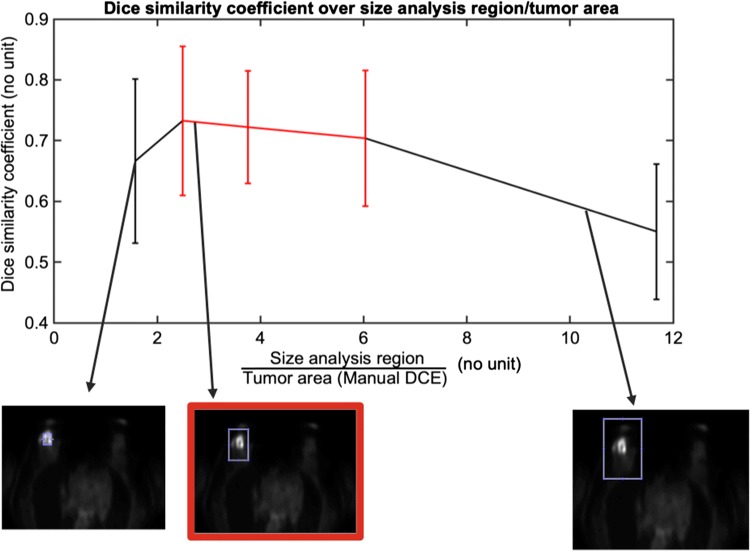
Dice similarity coefficient (cohort mean ± std) for Gaussian mixture model ROIs compared against manual dynamic contrast-enhanced (DCE) ROI. Indicated in red, the best results were given when the analysis regions were cropped at approximately 2–6 times the tumor area, and this was used in the study. This sensitivity would need to be accounted for in an automated cropping routine, as well as some sort of quality metric on the resulting ROI

Benefits of a data-driven, semi-automated GMM–PET approach include reduced radiologist workload, faster segmentation processes, and reduced interobserver variability [47]. The major advantage of PET/MRI systems, exploited here, is intrinsic registration of simultaneously acquired PET and MR data, giving voxel-level correspondence, without additional registration processes that generally involve user input or additional processing [48]. Further optimization of GMM–PET might include automated data-driven initialization and number of classes, with the goal of entirely eliminating manual input [49]. We illustrate the influence of lesion-region initialization, for which automation could be developed, in Appendix (Fig. 8). The number of voxels within the lesion is also a factor that will affect the performance of the GMM method, and is thus intrinsically tied to image resolution, and will ultimately limit use in smaller lesions.

One limitation to this methodology is that increased tracer uptake is less specific to disease than manual DCE [50], potentially introducing errors in the GMM–PET classification if not accounted for by appropriate selection of tumor-containing region. In addition, while the analysis in this study explicitly did not consider patient response from the clinical viewpoint, as well as other clinically relevant data such as hormone status, the variety of treatments and responses provided a suitably large range of situations in which to test the GMM–PET methodology, including cases where response included an almost total loss of detectable disease.

## Conclusion

The potential implications of improved imaging technology in breast cancer are large, and PET/MRI is a unique tool to investigate links between increased metabolism (PET), perfusion (DCE), and decreased diffusion (DWI), without additional scan time or registration errors. The current findings show that PET/MRI, using a semi-automated GMM segmentation strategy, yields tumor area and mean ADC value estimates that can replicate today’s gold standard of tumor definition of manual DCE from MRI. Furthermore, the GMM–PET method also captures tumor changes associated with response to neoadjuvant chemotherapy, which supplements today´s gold standard which is manual DCE in the neoadjuvant setting. The potential benefits include a broader assessment of morphological and metabolic changes to guide clinical decisions regarding tumor operability, and thus to ensure a high probability of complete tumor regression, and subsequent cancer cure.

## Appendix

See Figs. 7 and 8.
