# Correction to: Semi-automatic segmentation from intrinsically-registered 18F-FDG–PET/MRI for treatment response assessment in a breast cancer cohort: comparison to manual DCE–MRI

**DOI:** 10.1007/s10334-019-00789-5

**Published:** 2019-10-29

**Authors:** Maren Marie Sjaastad Andreassen, Pål Erik Goa, Torill Eidhammer Sjøbakk, Roja Hedayati, Hans Petter Eikesdal, Callie Deng, Agnes Østlie, Steinar Lundgren, Tone Frost Bathen, Neil Peter Jerome

**Affiliations:** 1 Department of Circulation and Medical Imaging, NTNU, Norwegian University of Science and Technology, Trondheim, Norway; 2 Department of Physics, NTNU, Norwegian University of Science and Technology, Trondheim, Norway; 3 Department of Radiology and Nuclear Medicine, St. Olav’s University Hospital, Trondheim, Norway; 4 Department of Clinical and Molecular Medicine, NTNU, Norwegian University of Science and Technology, Trondheim, Norway; 5 Department of Oncology, St. Olav’s University Hospital, Trondheim, Norway; 6 Section of Oncology, Department of Clinical Science, University of Bergen, Bergen, Norway; 7 Department of Oncology, Haukeland University Hospital, Bergen, Norway

## Correction to: Magnetic Resonance Materials in Physics, Biology and Medicine 10.1007/s10334-019-00778-8

The original version of this article unfortunately contained a mistake in Fig. 6.

The corrected Fig. 6 is placed in the following page.

**Fig. 6:**
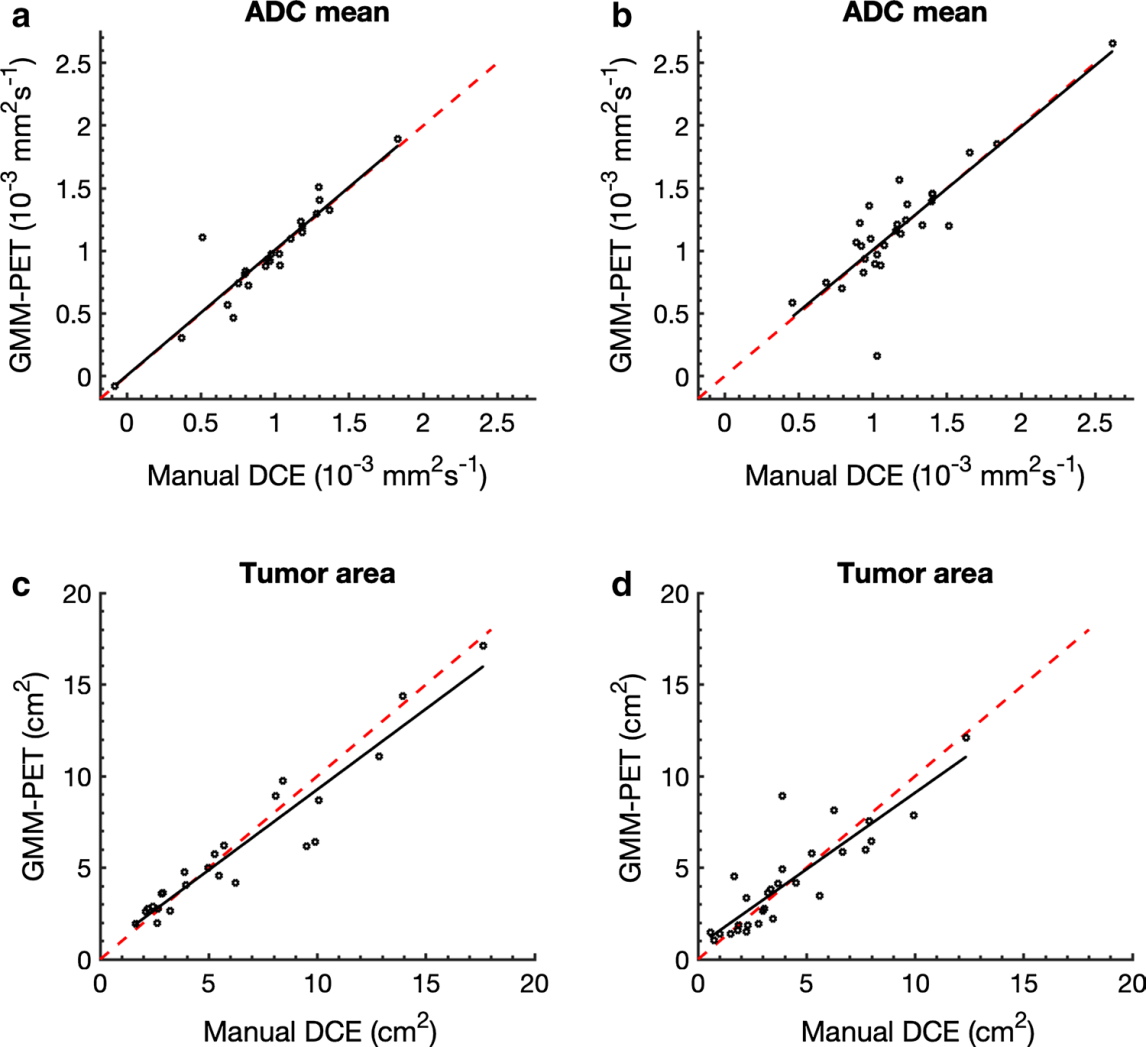
Relationship between the resulting metrics from manual DCE and GMM–PET for **a** ADC mean for untreated lesions (*r* = 0.866, *p *< 0.001) and **b** treated lesions (*r* = 0.895, *p *< 0.001) and m tumor area from **c** untreated (*r* = 0.870, *p *< 0.0001) and **d** treated (*r* = 0.928, *p *< 0.001) lesions. Red identity lines included show that area from GMM–PET is slightly smaller than from manual DCE

